# COVID-19 Vaccine Acceptance During Pregnancy, Women’s Views, and Influencing Factors

**DOI:** 10.7759/cureus.34039

**Published:** 2023-01-21

**Authors:** Keya Kalra, Ramanjeet Kaur, Priyanka Gupta, Atul K Sood

**Affiliations:** 1 Obstetrics and Gynecology, North Delhi Municipal Corporation (DMC) Medical College and Hindu Rao Hospital, New Delhi, IND; 2 Obstetrics and Gynecology, Hindu Rao Hospital, New Delhi, IND

**Keywords:** influencing factors, acceptance, vaccination, pregnant women, covid-19 vaccination

## Abstract

Background: COVID-19 vaccination is advised for pregnant women all over the world; however, vaccine acceptance is variable across the globe.

Methods: This study was conducted by enrolling 292 antenatal women attending the outpatient department (OPD) in a tertiary care hospital in Delhi, India, between August 1, 2022 and October 31, 2022, with the aim to determine the acceptability of vaccination against COVID-19 disease in pregnancy, women's views regarding the vaccine and to ascertain reasons for acceptance or denial during pregnancy.

Result: Of the 292 pregnant women who completed the questionnaire, 39.73% had received both doses of vaccination against COVID-19 disease before pregnancy, and 39.04% had received a single dose. Twenty-one percent of women did not receive any vaccine. Eighty-four percent of the unvaccinated and 35% of the women with a single dose of the vaccine refused further doses during pregnancy. The most common concern was the fear that the vaccine might cause harm to the fetus (35.3%), followed by the fear of vaccine-related reactions (25.4%). Also, 14.6% of women feared that the vaccine might cause them to abort the ongoing pregnancy. Thirteen percent of women stated their intent to receive the vaccine after they were informed regarding its safety and requirement. No difference was found in the acceptability of the vaccine based on the educational status of women or on the monthly income of the families.

Conclusion: Adequate communication regarding safety information on COVID-19 vaccines is a must for pregnant women and their families to provide reassurance about the need and safety of the vaccines. It would facilitate making informed decisions and is likely to be helpful in increasing vaccine acceptance.

## Introduction

It has been reported that SARS-CoV-2 infection in pregnant women is associated with increased risk for intensive care unit admission, mechanical ventilation, extracorporeal membrane oxygenation, and death as compared to complications seen in infected non-pregnant women [1-3]. In addition, a higher rate of adverse obstetric outcomes such as preeclampsia, thromboembolism, preterm birth, and stillbirth are also reported with SARS-CoV-2 infection [4,5].

Various studies have demonstrated that there is no increased risk of complications in the ongoing pregnancy or during delivery in vaccinated pregnant women when compared to non-vaccinated pregnant women [6-10]. Vaccinated pregnant women showed a significantly lower risk of SARS-CoV-2 infection, similar to the vaccinated general population [11]. In addition, evidence suggests that maternal vaccination during the third trimester has a potential protective effect on neonates, probably through placental immune transfer [12,13]. Guidelines from the Centre for Disease Control and Prevention (CDC), strongly recommend pregnant women receive vaccination, as well as a booster dose against COVID-19 disease [14]. Although vaccine hesitancy during pregnancy is not new, vaccination against COVID-19 disease in pregnancy faced difficulty due to a lack of sufficient evidence regarding its safety, and due to the reported side effects in the media [15].

## Materials and methods

Study population and recruitment

This cross-sectional observational study was conducted between August 1, 2022 and October 31, 2022, at North DMC Medical College and Hindu Rao Hospital, a tertiary care center in Delhi, India. Pregnant women attending antenatal OPD with any duration of pregnancy, consenting to the survey were recruited. The duration of pregnancy was calculated from the last menstrual period (LMP) or the first-trimester ultrasound if the dates were not available. Written questionnaires were distributed to those willing to enroll in the survey. Informed written consent was obtained from all the participants. Information provided by the participants was voluntary.

Survey questionnaires

The questionnaire for pregnant women was prepared by a team of obstetricians. A pilot survey involving pregnant women was conducted to ensure questionnaire comprehensiveness and changes were made accordingly. The survey included questions about maternal demographics and socioeconomic characteristics including age, comorbidity, duration of pregnancy, parity, educational status, whether social support was available, and status of childhood vaccination amongst others. The inclusion criteria were antenatal women willing to participate in the survey. Those women who did not desire to participate were excluded from the study. The participants were asked 15 questions regarding demographic details and vaccine-related information. The questionnaire contained the following questions amongst others: (1) Whether they were aware of COVID-19 disease and what were the information sources? (2) Did they have COVID-19 infection in the past? If yes, then details of its severity were sought, (3) Whether they had any knowledge about vaccination against COVID-19 disease and the sources of the information?, (4) Whether they have received the vaccine against COVID-19 disease. If immunized, the gestational age at vaccination, details regarding the type and dosage of the vaccine received were noted, (5) their reasons for refusing the vaccine were enquired, (6) inquire from the non-immunized pregnant women about their willingness to get vaccinated.

All data analysis was performed using SPSS version 25. All variables were categorical data, which were expressed as counts and percentages, and compared using the Chi-square test between vaccinated and unvaccinated groups in univariate analyses. Statistical significance was set at p < 0.05.

## Results

Out of all women attending antenatal clinics between August 1, 2022 and October 31, 2022, 292 agreed to participate in this survey. The demographic characteristics of these women are summarized in Table 1.

**Table 1:** Sociodemographic data of the study participants.

Parameter	_Mean±SD/ Median/%_
Age(years)	25.75 ± 4.13
Parity	2
Number of people in the household	6
Income/month (Indian rupees, INR)	₹18082.19 ± ₹14162.63
Education status	
Illiterate	8.9%
Primary school	8.9%
Secondary school	50.7%
Graduate	31.5%

Most women were primigravida (45%) and the common age group was between 20 and 30 years. The majority were literate, among them, 31.5%, were graduates and 50.7% had only secondary school education. Only a minority of the surveyed women were illiterate (8.9%). Seventy-six percent of women were living in joint families and 83.3% had social support in the city. Family income was between rupees 10,000-15,000 (2SD). Of the 292 pregnant women who completed the questionnaire, 39.73% had received both doses of vaccine against COVID-19 disease before pregnancy, and 39.04% had a single vaccine dose. Twenty-one percent of women did not receive any kind of COVID-19 vaccine. Eighty-four percent of the unvaccinated and 35% of the women with a single dose of vaccine refused further doses during pregnancy. The most common concerns regarding vaccination were the fear of vaccine causing harm to the fetus (35.3%), fear of vaccine-related relations (25.4%), and the risk of abortion following vaccination (14.6%) (Table 2). Thirteen percent of women stated their intent to receive the vaccine after being informed about its need and safety. Results of this study show that there is no difference in the acceptability of the vaccine based on the educational status of the women, nor did the monthly income of the family have any influence on the acceptance rates (Tables 3, 4).

**Table 2:** Reasons for refusing the COVID-19 vaccine.

^Reasons for refusal^	^No. of pregnant women (%)^ ^(N=102)^
^Afraid of vaccine^	^4 (3.9%)^
^Risk of vaccine complications/concern about its safety^	^26 (25.4%)^
^Risk of abortion^	^15 (14.7%)^
^Vaccine might harm the baby ^	^36 (35.3%)^
Family members have hesitancy toward the vaccine	^8 (7.8%)^
Ignorance/lack of knowledge	^13 (12.7%)^

 

**Table 3:** Acceptability of vaccine based on education status. The chi-square statistic is 4.5. The p-value is 0.1 (not significant).

	Illiterate (n)	Primary education (n)	Secondary education and above (n)
Women accepting vaccine	12	18	160
Women refusing vaccine	14	8	80

**Table 4:** Acceptability of vaccine based on income status. The chi-square statistic is 3.5044. The p-value is 0.06 (not significant) Below poverty line (BPL) in Urban India is defined as income below 1,286 Indian rupees/month ($0.52 per day).

	BPL	Above BPL	Total
Women accepting vaccine	12	178	190
Women refusing vaccine	13	89	102
Total	25	267	292

## Discussion

COVID-19 infection, deemed a pandemic by World Health Organization on March 11, 2020 [16], continues to affect a substantial number of people around the world. First detected in Wuhan, China in December 2019, it is an acute viral illness caused by the SARS-CoV-2 virus. Disease severity is varied from being asymptomatic in some to acute respiratory distress syndrome (ARDS) in others. Various studies have linked COVID-19 disease with complications in pregnancy such as pre-eclampsia, preterm labor, stillbirth, and thromboembolism [3-5]. Also, an increased risk of cesarean delivery has been reported [4].

The first confirmed case of COVID-19 disease in India was reported in Thrissur, Kerala on January 27, 2020. Since then, exhaustive efforts have been used to curtail the spread of infection. The evolution of the virus due to mutations, high infectivity, and mental fatigue has rendered preventive measures like social distancing, masks, and hand hygiene ineffective. Efforts to develop the vaccine against coronavirus started as soon as it began to sweep around the world. Two vaccines came early into circulation in India: Covishield and Covaxin. Both of these vaccines did not have clinical trials that included pregnant women initially. The vaccines which were being used in other parts of the world also had limited data regarding safety in pregnancy during the initial vaccination drive.

India began the administration of vaccines against COVID-19 disease on January 16, 2021. The first rollout involved healthcare workers and frontline workers including police, paramilitary forces, sanitation workers, and disaster management volunteers. It was then made available to those above 60 years of age. First May 2021 onward, it was extended to all Indian residents above 18 years of age, following the tragedy witnessed during the second wave of Covid-19 disease in India [17]. With accumulating data on the effectiveness of vaccines and the severity of COVID-19 disease during pregnancy, various international societies like the Royal College of Obstetrics and Gynecology (RCOG) recommended vaccination during pregnancy. Accepted by the Government of India on July 2, 2021, vaccination against COVID-19 disease is strongly recommended for all women who are pregnant or delivered or those who want to conceive.

Apprehension to get vaccinated during pregnancy arises from the fear for the baby and self. Top it with limited data regarding the safety of vaccines in pregnancy and local cultural beliefs, the rates of vaccination against COVID-19 disease in pregnancy are inconsistent around the globe [8-10]. Fear to vaccinate due to unknown fetal effects is the commonest reason for vaccine hesitancy. The present study was designed to determine the acceptance of the vaccines against COVID-19 disease among pregnant women visiting a government hospital in the capital of the country.

Our study reported a relatively high acceptance of COVID-19 vaccination as compared to a study done in Ankara City Hospital, Ankara, Turkey [18], which reported low acceptance of the vaccine due to a lack of data about safety in the pregnant population. Similar results of low acceptance were found in a cohort study of pregnant women who gave birth at St George's University Hospitals National Health Service Foundation Trust, London, United Kingdom, between March 1, 2020 and July 4, 2021 [19]. The vaccine acceptor group in our study thought they were informed adequately about the COVID-19 vaccine compared to the vaccine refusal group (P < 0.05). Media resources were their primary source of information. One of the main reasons for the higher acceptance of our study was fear due to high morbidity and mortality, as experienced during the second wave of COVID-19 disease in Delhi in the months of April-June 2021, which preceded our study. Moreover, our study enrolled women in tertiary care in the capital, and at a much later stage of vaccination coverage. Another reason for greater acceptance was the gradual rollout of the vaccine and massive media coverage regarding the effectiveness and essentiality of the vaccine. This reflects that public information sources are essential to ensure vital information reaches all populations of society.

We found no difference in the acceptability of the vaccine on basis of the educational status of the women (Table 3). The monthly income of the families also did not play any role in the acceptance rates of the vaccine as it was made freely available at various government healthcare facilities (Table 4). This reinforces the success of mass media coverage and an effective health policy strategy during the pandemic period.

Fear of the vaccine causing unknown fetal effects were the most common reason for vaccine hesitancy in our study, followed by fear of complications resulting from the vaccine. Influenza vaccination recommended during the 2009-10 influenza breakout in the United Kingdom faced similar apprehensions among pregnant women [20]. Lack of trust and doubt over the efficacy of the vaccine is the main reasons for refusal. Vaccination against tetanus has wider acceptability and no antenatal woman refused the vaccine. All the women knew that they had to receive tetanus vaccination during pregnancy as they understood that the tetanus vaccination resulted in decreased fetal and maternal morbidity and mortality. We feel that the long years of vaccination against tetanus have inculcated a sense of trust in the vaccine, with the recipient population understanding the need for vaccination. The success of tetanus vaccination in pregnancy is a very good example stressing the importance of having effective strategies for vaccination as a health policy.

Our study enrolled women in a tertiary care center, in the capital of the country and at a much later stage of vaccination coverage, which probably resulted in higher acceptance rates. The study group lacked participation by the disease-affected women and the rural population, which may have some influence on the acceptance rates of vaccination. The main strength of our study is that, to the best of our knowledge, this is the first such study done in our country to assess the vaccination acceptance against COVID-19 disease, which points out that good vaccination policy and massive media coverage both play an important role for good acceptance rates.

## Conclusions

With the COVID-19 disease still being a major health issue worldwide, vaccines play a major role in decreasing the burden on the already overburdened healthcare systems. Higher acceptance of the vaccine in this study was likely due to the fear of the disease, easy accessibility of the vaccine, well-informed public, and faith in the indigenous vaccine due to positive media coverage. Thus, educating pregnant women regarding the safety of vaccines provides reassurance about the need for vaccination and builds trust. It would facilitate in making informed decisions and is likely to be helpful for increasing vaccine acceptance further. 
